# Willow (*Salix* spp.) bark hot water extracts inhibit both enveloped and non-enveloped viruses: study on its anti-coronavirus and anti-enterovirus activities

**DOI:** 10.3389/fmicb.2023.1249794

**Published:** 2023-11-08

**Authors:** Dhanik Reshamwala, Sailee Shroff, Jaana Liimatainen, Jenni Tienaho, Mira Laajala, Petri Kilpeläinen, Anneli Viherä-Aarnio, Maarit Karonen, Tuula Jyske, Varpu Marjomäki

**Affiliations:** ^1^Department of Biological and Environmental Sciences, Nanoscience Center, University of Jyväskylä, Jyväskylä, Finland; ^2^Natural Resources Institute Finland (Luke), Helsinki, Finland; ^3^Natural Chemistry Research Group, Department of Chemistry, University of Turku, Turku, Finland

**Keywords:** antivirals, nature-based enteroviruses, coronaviruses, *Salix* spp., broad-spectrum

## Abstract

**Introduction:**

Recurring viral outbreaks have a significant negative impact on society. This creates a need to develop novel strategies to complement the existing antiviral approaches. There is a need for safe and sustainable antiviral solutions derived from nature.

**Objective:**

This study aimed to investigate the antiviral potential of willow (*Salix* spp.) bark hot water extracts against coronaviruses and enteroviruses. Willow bark has long been recognized for its medicinal properties and has been used in traditional medicines. However, its potential as a broad-spectrum antiviral agent remains relatively unexplored.

**Methods:**

Cytopathic effect inhibition assay and virucidal and qPCR-based assays were used to evaluate the antiviral potential of the bark extracts. The mechanism of action was investigated using time-of-addition assay, confocal microscopy, TEM, thermal, and binding assays. Extracts were fractionated and screened for their chemical composition using high-resolution LC-MS.

**Results:**

The native *Salix* samples demonstrated their excellent antiviral potential against the non-enveloped enteroviruses even at room temperature and after 45 s. They were equally effective against the seasonal and pandemic coronaviruses. Confocal microscopy verified the loss of infection capacity by negligible staining of the newly synthesized capsid or spike proteins. Time-of-addition studies demonstrated that *Salix* bark extract had a direct effect on the virus particles but not through cellular targets. Negative stain TEM and thermal assay showed that antiviral action on enteroviruses was based on the added stability of the virions. In contrast, *Salix* bark extract caused visible changes in the coronavirus structure, which was demonstrated by the negative stain TEM. However, the binding to the cells was not affected, as verified by the qPCR study. Furthermore, coronavirus accumulated in the cellular endosomes and did not proceed after this stage, based on the confocal studies. None of the tested commercial reference samples, such as salicin, salicylic acid, picein, and triandrin, had any antiviral activity. Fractionation of the extract and subsequent MS analysis revealed that most of the separated fractions were very effective against enteroviruses and contained several different chemical groups such as hydroxycinnamic acid derivatives, flavonoids, and procyanidins.

**Conclusion:**

*Salix* spp. bark extracts contain several virucidal agents that are likely to act synergistically and directly on the viruses.

## 1. Introduction

The emergence of viral outbreaks leading to epidemics and pandemics causes a huge strain on the global economy and public health. The recent pandemic caused by severe acute respiratory syndrome coronavirus 2 (SARS-CoV-2) has been a catastrophic event, and as of June 2022, it has caused over 6.9 million deaths worldwide (WHO, 2022). SARS-CoV-2 belongs to the β-coronavirus genus, which also includes SARS-CoV and Middle East respiratory syndrome coronavirus (MERS-CoV). It is an enveloped, positive-sense single-stranded RNA (+ssRNA) virus with a diameter of 60–140 nm (Pizzato et al., 2022). Transmission of SARS-CoV-2 occurs through aerosol, the fecal-oral route, and surface contamination (Karia et al., 2020; Zhou et al., 2021) to cause lower respiratory tract infections. The group of beta coronaviruses (HCoVs) also includes several seasonal coronaviruses that cause the common cold. HCoV strain OC43 is responsible for 15–30% of mild upper respiratory tract infections in humans (Gaunt et al., 2010). Both belong to the β-coronavirus genus and are closely related genetically (Lu et al., 2020). Moreover, both of these viruses replicate in the human respiratory epithelium and spread via aerosols and droplets (Kutter et al., 2018). Enteroviruses are also positive-sense single-stranded RNA (+ssRNA) viruses, but in many ways different from coronaviruses: They are non-enveloped, much smaller in size (30 nm in diameter), and stay very stable and infectious on surfaces and in the environment. They are responsible for causing acute infections such as flu, meningitis, pancreatitis, and myocarditis. They are also associated with chronic infections like type 1 diabetes (Nekoua et al., 2022), asthma, and chronic obstructive pulmonary disease (COPD) (Kurai et al., 2013). Enteroviruses include several serotypes that infect through the fecal–oral route but also viruses that infect through the respiratory route, such as rhinoviruses and enterovirus D68, also called the “new polio” (Cassidy et al., 2018).

Antiviral agents, such as vaccines, drugs, and virucides, help in reducing viral transmission. Virucides are used to reduce the viral load on the surface and in the environment. They are used as disinfectants for surface sterilization of biological and medicinal products. Additionally, they have been used to inactivate viruses in foodstuffs, detergents, and cosmetics (Galabov, 2007). However, the majority of the virucides are chemical disinfectants, which are hazardous in nature and cause environmental contamination. In addition, they cause side effects on human health, such as skin irritation. Moreover, non-enveloped viruses like enteroviruses are largely resistant to chemical disinfectants (Chan and Abu Bakar, 2005; Sauerbrei and Wutzler, 2010). Even though vaccines are an effective weapon against virus infection, it is not feasible to develop a vaccine against all the enteroviruses. In addition, the process of vaccine development and approval also takes time. Currently, there are no clinically approved drugs for enteroviruses. Thus, there is a great need to find broadly acting antiviral agents that would lower the infectivity of viruses around us and that could complement the vaccines and drugs in the combat against viruses. Natural products are a rich source of bioactive compounds. Out of 1,881 approved drugs from the start of January 1981 to the end of September 2019, a total of 41.8% are either biological macromolecules, unaltered natural products, botanical drugs, or natural product derivatives. If synthetic products mimicking natural compounds are also considered, the share increases to 64.3% (Newman and Cragg, 2020). Various natural products have been reported to exhibit antiviral activity, and they are an interesting source of novel antivirals because of their availability, tolerability, and expected low side effects (Kumar and Pandey, 2013; Goh et al., 2020). As an example, flavonoids are a diverse group of plant secondary metabolites known for their antioxidant, anti-inflammatory, anticarcinogenic, and other therapeutic properties (Kumar and Pandey, 2013; Panche et al., 2016). While often the mechanism of antiviral action remains unknown, natural products have been reported to interact with the viral life cycle by either targeting viral entry, replication, assembly, or release (Lin et al., 2014; Linnakoski et al., 2018).

In our previous study, we showed that *Salix* bark hot water extracts are highly effective against non-enveloped enteroviruses (Coxsackie virus A9) and not cytotoxic in the used concentrations (Tienaho et al., 2021). Interestingly, none of the tested reference compounds, such as triandrin, salicin, salicylic acid, or picein, showed antiviral activities, suggesting that the bioactive properties of *Salix* clone bark extracts could be due to the synergistic effects of different bioactive agents such as tannins and other polyphenols. In the present study, *Salix* bark hot water extracts were tested for their antiviral activity against HCoV-OC43 and SARS-CoV-2, and their mechanism of action was elucidated against the coronaviruses and for the previously tested enteroviruses. Bark extracts from most of the willow clones tested showed antiviral potency against both viruses by having a direct effect on the virus particles. The extracts caused clustering of both the viruses but halted infection in different ways for non-enveloped and enveloped viruses: through the increased stability of enteroviruses structure, but through the compromised structure of coronaviruses.

## 2. Methods

### 2.1. Cells

Human alveolar basal epithelial adenocarcinoma (A549), Vero E6, and human lung fibroblasts (MRC-5) cells were obtained from the American Type Culture Collection (ATCC) (Manassas, VA, USA). The A549 cells were propagated in Dulbecco's modified Eagle medium (DMEM) (Gibco, Paisley, UK), whereas MRC-5 and Vero E6 cell lines were grown in Eagle's minimum essential medium (MEM) (Gibco, Paisley, UK). Both MEM and DMEM were supplemented with 10% fetal bovine serum (FBS, Gibco, Paisley, UK), 1% l-GlutaMAX (Gibco, Paisley, UK), and 1% antibiotics (penicillin/streptomycin) (Gibco, Paisley, UK) and stored in a humidified 5% CO_2_ incubator at 37°C.

### 2.2. Viruses

Coxsackievirus B3 (CVB3; Nancy strain) and Coxsackievirus A9 strain (CVA9; Griggs strain) were obtained from ATCC. They were produced and purified as described before (Myllynen et al., 2016; Ruokolainen et al., 2019), with one exception of adding 0.1% (v/v) TWEEN^®^ 80 (Sigma-Aldrich, Steinheim, Germany) during the freeze–thaw cycle. Seasonal human coronavirus HCoV-OC43 (ATCC) was used as a crude or purified preparation. SARS-CoV-2 (SARS-CoV-2/Finland/1/2020) was isolated from the first COVID-19 patient in Finland (Haveri et al., 2020).

### 2.3. *Salix* sample collection and preparation

The *Salix* clone sample collection and extraction have been previously published by Tienaho and colleagues (Tienaho et al., 2021). For this study, 16 *Salix* clones were chosen (Table 1). Out of these, 12 were examples of widely distributed native Finnish species: three *S. myrsinifolia* Salisb. clones, four *S. phylicifolia* L. clones, and three natural and two artificial hybrids of these. Four clones originated from the Swedish willow breeding program. Detailed information on the growing media, growth coordinates, and handling was published earlier (Tienaho et al., 2021). In brief, the native willow clones were harvested in May 2019 as 2-year-old 1–1.5 m in length coppice with 0.5–2 cm diameter at the base, and commercial willow samples were grown by Carbons Finland Ltd. and cut down in March 2019 when 3-year-old and the 3-m-long sample shoots were cut to ca. 40-cm-long pieces. The harvested willows were packed separately in plastic bags and immediately frozen at −20°C before handling. Two shoots of each willow were debarked 50 cm from the base and pooled. The obtained bark was cut into small pieces, frozen at −80°C, freeze-dried before being ground with a Moulinex grinder into 1- to 2-mm pieces, and kept frozen at −80°C until extracted using an ASE-350 accelerated solvent extractor (Dionex, Sunnyvale, CA, United States). The bark sample (1 g) was placed in a stainless-steel extraction vessel (22 ml). The sample was then extracted three times for 15 min with hot water at 90°C, and the extract was stored at −20°C before further analyses. In addition, clone 16 was extracted in a larger-scale extraction vessel. For this experiment, willow clone 16 stand was cut down in October 2020 by Carbons Finland Ltd. from a willow bank that was partly harvested in 2018 and 2019. The shoots were debarked immediately and frozen at −20°C. The bark was ground with a Kamas cutting mill with a 2-cm sieve and extracted in a 2-L stirring reactor (Polyclave, Büchi, Switzerland) with hot water (80°C) (Tienaho et al., 2021). Total dissolved solids (TDS) of extracts varied from 4.1 to 8.6 mg/ml.

**Table 1:** *Salix* spp. clones used in this study.

**Sample number**	**Species**	**Type**	**Clone**
1	*S. myrsinifolia*	Native	E6682
2	*S. myrsinifolia*	Native	E6771
3	*S. myrsinifolia*	Native	E6948
4	*S. phylicifolia*	Native	E6666
5	*S. phylicifolia*	Native	K2191
6	*S. phylicifolia*	Native	K2218
7	*S. phylicifolia*	Native	K2277
8	*S. myrsinifolia* × *phylicifolia*	Native hybrid	K2183
9	*S. myrsinifolia* × *phylicifolia*	Native hybrid	K2269
10	*S. myrsinifolia* × *phylicifolia*	Native hybrid	K2341
11	(K2183 *S. myrs*. × *phyl*.) × S15136 *S*.gmelinii^*^	Artificial hybrid	V7545
12	(K2183 *S. myrs*. × *phyl.)* × P6011 *S*.gmelinii^*^	Artificial hybrid	V7546
13		Commercial clone	Scherenee
14		Commercial clone	Tordis
15		Commercial clone	Tora
16		Commercial clone	Klara

^*^S. gmelinii Pall. is former S. dasyclados Wimm (Väre et al., 2021).

### 2.4. Extraction and fractionation of willow stem

For a pilot-scale extraction, 1-year-old shoots of the commercial willow variety, Klara, were harvested by Carbons Finland Ltd. The growing site was a peat field at Aitomäki, Kouvola, in south-eastern Finland (N60°52′0.01″ E26°41′60.00″). The shoots were cut in September 2022 and immediately transported to the piloting site in Bioruukki, Espoo, Finland. Whole willow shoots, without prior debarking, were milled with a shredder (Viking GE 150, VIKING GmbH). Shredded willow was collected into a bag and stored at −30°C before the extraction. The moisture content of the shredded material was 52.5 wt%, determined by oven drying at 105°C overnight.

For hot water extraction, a 64.8 kg batch (34.0 kg o.d.) of freshly shredded willow was added into a 300 L extraction system (Kilpeläinen et al., 2014). Water was pre-heated to 135°C to obtain the targeted 90°C extraction temperature. The average temperature during the extraction was 92°C, the pressure during the extraction was 10 bars, and the extraction time was 60 min. Extract (216 kg) was collected into an intermediate bulk container. Extract's total dissolved solids (TDS) was 1.19 wt%, indicating that 76 mg/g (o.d.) of the original shredded willow sample was obtained. Finally, the extract was lyophilized.

The fractioning of the stem extract was performed according to previously reported methods with some changes (Salminen and Karonen, 2011; Tian et al., 2018). 5 g of willow extract was dissolved in 50 ml of water using an ultrasonic bath. Extract was applied into a Sephadex LH-20 column (dimensions of the resin bed: 5.0 cm i.d. × 21 cm) and eluted successively with water (500 mL), aqueous ethanol (20, 40, 60, and 80% ethanol, 500 mL for each), and aqueous acetone (30, 50, and 70% acetone, 500 mL for each, except 700 for 70% acetone). Eight fractions were collected. Fractioning was repeated two times, and the equivalent fractions were combined. Fractions were concentrated by rotary evaporation at 45°C and finally lyophilized.

### 2.5. Commercial substances and samples

Commercial substances were used as references in the antiviral screening assays. Salicin and picein (purity >98%) were purchased from Merck Life Science Oy. Salicylic acid (purity >99%) was obtained from VWR Chemicals, and triandrin (purity 85%) was obtained from Molport EU. Additionally, Salixin Organic Powder (48^TM^) and Salixin Organic Extract (800NP^TM^) were supplied by Søren Fisker (Salixin A/S) and Pia Wikström (OY CELEGO AB) and were also tested for their antiviral efficacy along with the reference substances.

### 2.6. Antiviral activity assay

The screening of the bark extracts from 16 *Salix* clones to determine their antiviral activity against HCoV-OC43 was performed using the cytopathic effect (CPE) inhibition assay, modified from our previous study (Reshamwala et al., 2021). In brief, MRC-5 cells at a density of 15,000 cells/well were cultured in 100 μl of MEM supplemented with 10% FBS, 1% GlutaMAX, and 1% penicillin/streptomycin antibiotics on a 96-well flat-bottomed microtiter plate (Sarstedt, Numbrecht, Germany) for 24 h at 37°C. The next day, the virus was pre-treated with *Salix* bark extract (1% v/v) by preparing a virus–extract mix in 2% MEM and incubating it for 1 h at 34°C. The virus titer in the virus–extract mix was 2 × 10^4^ PFU/ml. A virus without the extract was used as a positive control, and a mock infection without the virus and extract was used as a negative control for the experiment. Reference substances and commercial samples mentioned above were also tested at different concentrations against the virus. Following this, the virus–extract mix was added to the cells (MOI of 0.1) for 2 h at 34°C. After the incubation, cells were aspirated, and fresh media was added. Finally, cells were incubated for 5 days at 34°C or until the cytopathic effect was observed. *Salix* bark extract was also tested against CVB3 using the CPE inhibition assay. The experiment was performed similarly as described in a previously published article (Tienaho et al., 2021). The only difference was the CVB3 titer and *Salix* extract amount, which was 2 × 10^6^ PFU/ml and 0.1% v/v, respectively, in the *Salix*–virus mix, and the final MOI was 10. Once the cytopathic effect was observed under the light microscope, cells were fixed and stained for 10 min using the CPE dye (0.03% crystal violet, 2% ethanol, and 3.5% formaldehyde). The stained viable cells were then washed two times with water, following which they were lysed using a lysis buffer (0.8979 g of sodium citrate and 1 N HCl in 47.5% ethanol). Finally, the absorbance of the viable cells in the 96-well plate was measured spectrophotometrically at 570 nm using the PerkinElmer VICTOR^TM^ X4 multilabel reader (PerkinElmer, Turku, Finland). The assay was performed two times independently.

### 2.7. Antiviral activity assay for SARS-CoV-2

Vero E6 cells at a density of 50,000 cells/well were cultured in 100 μl of MEM supplemented with 10% FBS, 1% GlutaMAX, and 1% penicillin/streptomycin antibiotics on a 96-well flat-bottomed microtiter plate for 24 h at 37°C. The following day, SARS-CoV-2 was pre-treated with 1% v/v of *Salix* bark extract of clone 5 or P-16 by preparing a virus–extract mix in 2% MEM and incubating it for 1 h at 34°C. Handling of the virus was carried out at the BSL-3 facility at the University of Helsinki, Finland. The virus titer in the virus–extract mix was 20 PFU/ml. After the incubation, the virus–extract mix was added to cells (MOI-0.00002) for 2 h at 34°C. Following the incubation, the cells were aspirated, fresh media was added, and they were incubated for 3 days at 34°C. Finally, the supernatant solution from the cells was collected and transferred to a new 96-microtiter plate for the extraction of viral RNA. The extraction was done using a Chemagic Viral RNA/DNA Kit (PerkinElmer, Turku, Finland). Once the viral RNA was extracted, we performed a real-time reverse transcriptase polymerase chain reaction (RT-qPCR) to qualitatively detect viral nucleic acid. This was performed using a SARS-CoV-2 RT-qPCR reagent kit (PerkinElmer, Turku, Finland). To compare the relative amounts of RNA in samples, we make use of the fact that 1 difference in Cq (cycle quantification) value means ~2 × difference in RNA amount. An equation (RNA difference = 0.9646e^0.6948x^, *x* is the difference in the Cq values between the mean of test samples and the mean of virus control] was deduced by using Cq differences down from 10 (10 cycle difference meaning ~1000 difference in relative RNA amount). This was used in our calculations to gain a value for RNA difference, of which a log value was then calculated to describe the difference in virus amounts.

### 2.8. Time and temperature assay

The time and temperature studies were performed using the CPE inhibition assay as described above. The only modification was in the incubation temperature (room temperature and 34°C) and time interval (45 s and 5 min) between the virus and *Salix* bark extracts. The room temperature (RT) monitored by the sensor was 21 ± 1°C.

### 2.9. Time-of-addition studies

Time-of-addition studies of the *Salix* bark extract were performed using the CPE inhibition assay as described above. For this assay, three modes of infection were designed. In the pre-infection mode, cells were incubated with the *Salix* bark extract (1% v/v) for 1 h at 34°C. After the incubation, cells were washed briefly on ice and then infected with HCoV-OC43 (MOI 0.01) for another 1 h at 34°C. Following the infection, cells were washed, and fresh media was added and incubated for 5 days at 34°C. In the co-infection mode, a mix of the virus (1.6 × 10^3^ PFU/ml) and the *Salix* bark extract (1% v/v) was prepared and added directly to cells for 1 h. Following the incubation, cells were aspirated, and fresh media was added and incubated for 5 days at 34°C. In the post-infection mode, cells were infected with HCoV-OC43 (MOI 0.01) for 1 h at 34°C. After the infection, the excess virus was removed by repeated washing. Then, media containing the *Salix* bark extract (1% v/v) was added (after 1 h) and the cells were incubated for 5 days at 34°C. The schematic showing the experimental design for time-of-addition studies is shown below. Virus control (without the *Salix* bark extract), *Salix* bark extract control (without virus), and mock infection were used as controls during each of the different modes of infection studied. Extracts from willow clones 5, 8, 10, and 16 (Table 1) were used for this assay. This experiment was performed two times independently.

### 2.10. Virucidal assay (endpoint dilution assay)

Quantification of the reduction in viral infectivity after treatment with the *Salix* bark extract was performed using a virucidal assay modified by Alvarez and colleagues (Álvarez et al., 2022). Briefly, MRC-5 cells were seeded at a density of 15,000 cells/well on the 96-well flat-bottomed microtiter plate and incubated for 24 h in 5% CO_2_ and 37°C. The next day, HCoV-OC43 (1:2 dilution) was mixed with *Salix* bark extracts of clone 8 or 10 (50% v/v) and incubated for 15 min at RT. A virus control with a similar amount of virus without the *Salix* bark extract was also incubated. After the incubation, the virus–extract mix was diluted 100 times using 2% MEM. Following this, we performed 10-fold serial dilutions (10 dilutions in total). Each dilution was added in replicates of eight to the cells and incubated for 5 days at 34°C. Following the incubation, the cells were stained with the crystal violet dye for 10 min to differentiate between the healthy and infected cells. Finally, the virus titers were calculated using the Reed–Muench method (Reed and Muench, 1938) and expressed as particle-forming units (PFU) per ml.

### 2.11. Particle stability thermal release assay (PaSTRy) for enterovirus

The PaSTRy assay was performed as described before (Martikainen et al., 2015). The assay is based on recording the temperature at which the viral RNA becomes accessible to Sybr Green II (SGII), and the emitted fluorescence is detected by the CFX Real-Time PCR instrument (Bio-Rad C100, Helsinki, Finland). A reaction mixture of 50 μl containing 1 μg of CVA9 and *Salix* bark extract of clones 5, 10, or 16 (10% v/v) in PBS were incubated for 1 h at 37°C. After the incubation, 10 × SGII (Invitrogen) diluted in double-distilled water (ddH_2_O) was added to the reaction mix and then aliquoted into a thin-walled PCR plate (Agilent, Amstelveen, Netherlands). The thermal cycler recorded the fluorescence in quadruple from 20 to 90°C with 0.5°C intervals. The fluorescence data output was extracted from the BioRad CFX manager (2.1 software, accessed on 1 March 2022) and processed in GraphPad PRISM. The relative fluorescence emission (RFU) was plotted as a function of temperature to obtain the melt curve, and the melting temperature could be determined from the melt peak, which was plotted using the derivative of the RFU as a function of temperature [d (RFU)/dT].

### 2.12. Negative staining for transmission electron microscopy

To visually understand the effect of the *Salix* bark extract compounds on the structure of enteroviruses and coronaviruses, we imaged the *Salix* extract-treated viruses under the transmission electron microscope (TEM) JEM-1400 (JEOl, Tokyo, Japan). Prior to sample preparation with the coronaviruses and enteroviruses, formvar-coated copper grids were glow discharged (EMS/SC7620 mini-sputter coater) for 30 s and placed on a parafilm inside a Petri dish. A reaction mixture containing four parts of HCoV-OC43 or CVA9 (OC43 and CVA9 stock infectivity: 7.43 × 10^9^ PFU/ml and 5.47 × 10^9^ PFU/ml, respectively) and one part of *Salix* bark extract of clones 5 or 10 were added onto the grid and mixed gently with a pipette. An untreated virus of the same amount was used as a virus control. The treated and untreated virus samples were incubated inside a Petri dish at RT for 15 min. Subsequently, the excess of the sample was blotted away using Whatman paper (Whatman 3 MM). The samples were stained with 5 μl of 1% (w/v) phosphotungstic acid for 10 s, and the excess stain was blotted away using Whatman paper. The samples were left to air dry in a grid box overnight before imaging with the TEM, equipped with a field emission gun and LaB_6_ filament, operating at a voltage of 80 kV in the BF-TEM imaging mode. The images were taken with a bottom-mounted Quemesa CCD camera with a resolution of 4008 × 2664 pixels.

### 2.13. Binding assay for coronaviruses

MRC-5 cells at a density of 70,000 cells/well were cultured in MEM supplemented with 10% FBS, 1% GlutaMAX, and 1% penicillin/streptomycin antibiotics in a 24-well flat-bottomed plate (Fischer Scientific) for 24 h in 5% CO_2_ and 37°C. The next day, 10% v/v of Clone 5 or 10 was pre-treated with the virus (6.98 × 10^6^ PFU/ml) for 1 h at 37°C. A virus control with the same amount of virus under the same conditions was also used. The pre-treated virus and virus control were then added to the MRC-5 cells at a MOI of 50. The virus was allowed to bind to the cells for 1 h on ice under rocking conditions. Post that, the media was taken out, and three gentle washes with 0.5% BSA/PBS, 5 min each, were given to remove any unbound viruses. After the last wash, the cells were detached from the bottom using buffer AVL from the RNA isolation kit (Qiagen, Hilden, Germany, ref. 52906), and the RNA was isolated. The isolated RNA was converted into cDNA using a Promega kit and reverse primer (5′-AATGTAAAGATGRCCGCGTATT) (Merck). The cDNA was amplified using the BioRad kit, reverse primer, forward primer (5′-TGTTAGGCCRATAATTGAGGAC) (Merck), and running it through the Touch Thermal Cycler (Bio-Rad C1000, Helsinki, Finland). The amplification steps were as follows: 10 min at 95°C, 40 cycles of 15 s at 95°C and 1 min at 50°C, 5 s at 72°C, 1 min at 95°C, followed by cooling at 12°C for 10 min.

### 2.14. Immunofluorescence labeling and microscopy

MRC-5 cells were seeded at a density of 8000 cells/well on the 96-well flat-bottomed microtiter plate (Fisher Scientific, Finland) and incubated for 24 h in 5% CO_2_ and 37°C. The next day, the virus was pre-treated with *Salix* bark extract (1% v/v) by preparing a virus–extract mix in 2% MEM and incubating it for 1 h at 34°C. The virus–extract mix was added to the cells (MOI of 50) for 1 h at 4°C or at RT, after which the excess virus was washed with PBS. Next, fresh 2% MEM was added, and infection was allowed to proceed at 34°C for 1 h or overnight before fixing with 4% paraformaldehyde for 30 min. The cells were then permeabilized with 0.2% Triton X-100. Following this, they were treated with primary antibodies: rabbit antibody against the S-protein of HCoV-OC43 (a kind gift from Professor Ilkka Julkunen, University of Turku, Finland), mouse J2 antibody against the dsRNA of the virus (Scicons, Hungary), and mouse tubulin antibody (Santa Cruz Biotechnology, USA).

*Salix* bark extract (1% v/v) was also studied for its effect on CVA9 using confocal microscopy. Here, the *Salix*–virus mix was prepared in a buffer (PBS with 2 mM MgCl_2_) and incubated for 1 h at 37°C. After the incubation, the mix was diluted with 10 × DMEM, then added to A549 cells (MOI of 100) and incubated for 6 h at 37°C before fixation as described above. The primary antibodies used were rabbit antibodies labeling the CVA9 capsid and mouse J2 against the dsRNA of the virus. After 1 h of incubation, cells were washed with PBS to remove excess primary antibody and then treated for 30 min with secondary antibodies: goat anti-rabbit Alexa 555 (Invitrogen Life Technologies, USA) or goat anti-mouse Alexa 488 (Invitrogen, Life Technologies, USA). Secondary antibodies were washed with PBS, and cell nuclei were stained with DAPI (Molecular Probes, Life Technologies, USA) in PBS.

Samples were imaged with a Nikon A1R confocal microscope. The imaging was carried out with the 40 × objective (NA 1.25), 405 nm diode laser, 488 nm multiline argon laser, and 561 nm sapphire laser. Laser power and detector amplification settings were optimized for each channel. Virus protein and dsRNA channels were adjusted according to the cell control to exclude antibody background. Images were visualized using the software Fiji2 (ImageJ). CellProfiler 4.2.1 was used to determine the number of infected cells in a sample. First, the nuclei were identified as primary objects using the Otsu thresholding method. Next, the infected cells were identified as secondary objects using the previously identified nuclei as a reference. The manual thresholding method was used to differentiate the background from the virus protein signal. Finally, the area and intensity of the secondary objects were measured, and the data were exported to Excel, where a threshold was set manually to differentiate infected from non-infected cells. Quantification was done to calculate the infection (%) by comparing the infected cells of the virus control with those of the test samples. At least 500 cells per sample were analyzed in the HCoV-OC43 and CVA9 assays.

### 2.15. Chemical characterization

Willow bark and stem extracts and fractions were analyzed by high-resolution LC–MS according to an earlier method (Karonen et al., 2021). Briefly, the UPLC-DAD-ESI-QOrbitrap-MS/MS instrument consisted of an Acquity UPLC system (Waters Corp.) coupled to a quadrupole-Orbitrap mass spectrometer (QExactiveTM, Thermo Fisher Scientific GmbH). The column was an Acquity UPLC BEH Phenyl (2.1 × 100 mm, 1.7 μm, Waters Corp.), and acetonitrile and 0.1% aqueous formic acid were used as eluents. The UV and MS data were acquired throughout the analysis. Negative ionization was used with a spray voltage of −3.0 kV and in-source collision-induced dissociation (CID) set at 30 eV. The mass range of orbitrap was m/z 150–2250 for the full scan.

### 2.16. Statistical analysis

Statistical analysis was performed using GraphPad Prism 6 (GraphPad Software, San Diego, CA, USA). Data are presented as mean + standard error (SEM). One-way ANOVA, followed by the Bonferroni test (^*^p < 0.05, ^**^p < 0.01, ^***^p < 0.001, and ^****^p < 0.0001), was used to assess the statistical significance of the differences between treated and untreated virus samples.

## 3. Results

### 3.1. Determining the broad-spectrum antiviral activity of *Salix* bark extract

**Figure 1:**
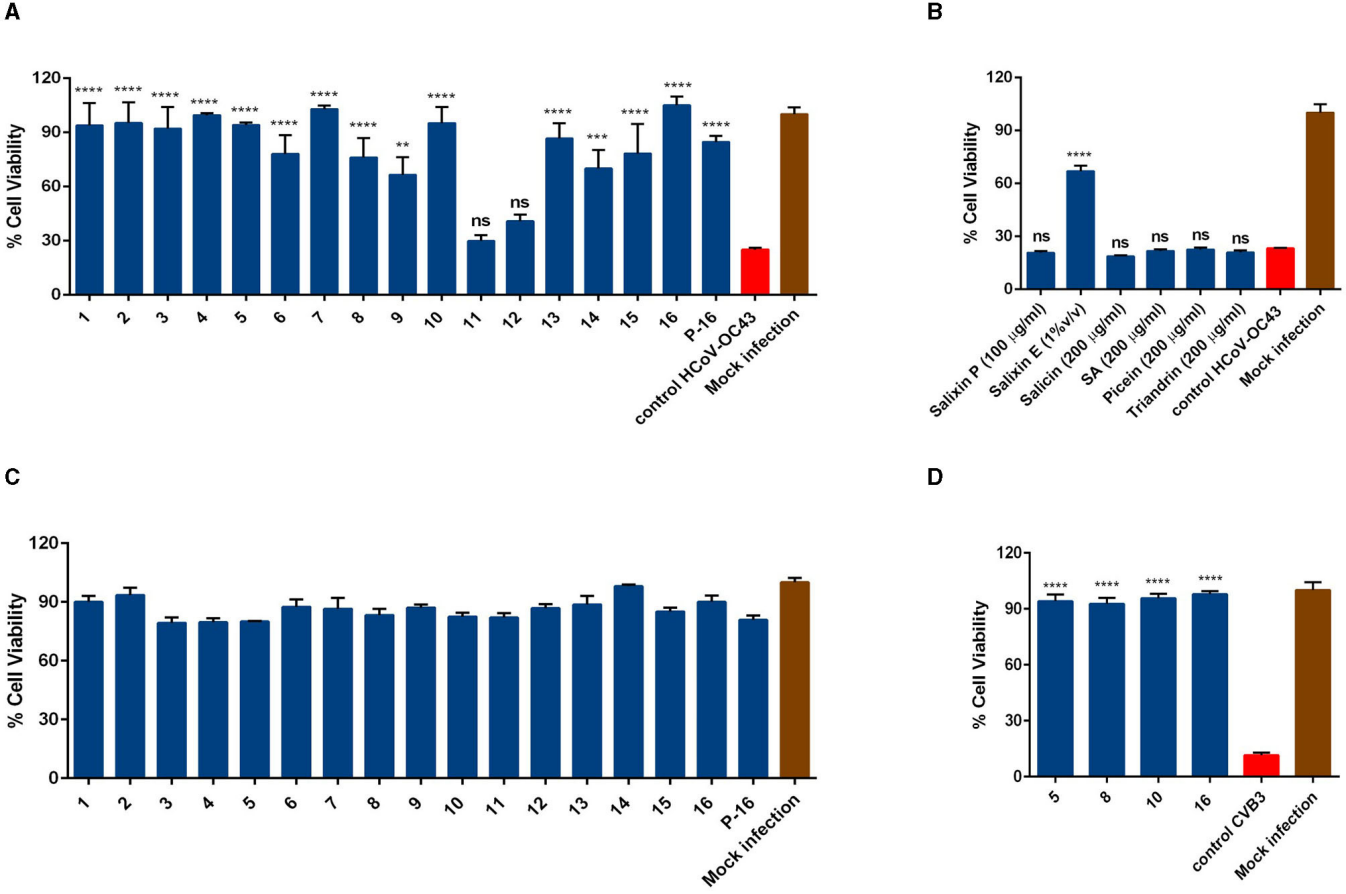
Testing **(A)** antiviral activity of bark extracts of *Salix* clones (1% v/v) **(B)** reference compounds (salicin, salicylic acid, picein, and triandrin) and Salixin organic powder and extract against HCoV-OC43 **(C)** Testing the cytotoxicity of bark extract of *Salix* clones (1% v/v) on MRC-5 cells **(D)** Testing the antiviral activity of *Salix* bark extracts of different clones (0.1% v/v) against CVB3. All the experiments were performed using the CPE inhibition assay. Virus control and test samples are normalized against the mock infection. The results are the mean of two independent experiments and are shown as average values + standard error of the mean (SEM). ^**^p_ANOVA_ < 0.01, ^***^p_ANOVA_ < 0.001, and ^****^p_ANOVA_ < 0.0001. ns, not significant; P-16, 2-L scale clone 16; Salixin P, Salixin Organic Powder 48^TM^; Salixin E, Salixin Organic Extract 800NP^TM^; SA, salicylic acid.

First, the antiviral potential of the *Salix* bark extracts was evaluated against the seasonal human coronavirus OC43 using the CPE inhibition assay. The virus was pre-treated with extract at 34°C for 1 h before being added to the cells. The bark extracts of all 16 *Salix* clones were tested at 1% v/v concentration. A virus sample without the extract was used as a positive control, and a mock infection without the virus and extract was used as a negative control for the experiment. The screening result showed that the bark extract of all the willow clones (except for 11 and 12) at 1% v/v concentration was able to protect the MRC-5 cells from HCoV-OC43 infection (Figure 1A). Research on *Salix* spp. bark extracts over the years has identified several key compounds that could play a major role in their pharmacological properties (Julkunen-Tiitto, 1985; Kammerer et al., 2005; Dou et al., 2018). Hence, we tested some of these reference substances (salicin, salicylic acid, picein, and triandrin) against the virus to understand if these substances contributed to the antiviral activity of the extract. However, none of the reference substances were able to block the virus infection, even if we had 10-fold higher concentrations of the reference compounds in comparison to the bark extracts (Figure 1B). We also tested Salixin Organic Powder (48^TM^) and Salixin Organic Extract (800NP^TM^) along with the reference compounds. Like the willow clones, Salixin Organic Extract showed antiviral activity against the virus, whereas the powder sample did not protect the cells from HCoV-OC43 infection. Cytotoxicity studies for bark extract of all 16 *Salix* clones (1% v/v) were also carried out similarly to the screening antiviral assay. From the result (Figure 1C), it was evident that the extract of none of the clones was toxic to the MRC-5 cells at the tested concentration. In our previous article (Tienaho et al., 2021), we had already shown the antiviral activity of the *Salix* bark extract against CVA9. We further wanted to assess whether the bark extracts of different willow clones are equally effective against another serotype of enterovirus. So, we tested some of the clones (5, 8, 10, and 16) against CVB3 and found that the bark extracts of all the clones tested were able to inhibit CVB3 infection and protect the A549 cells (Figure 1D). Thus, *Salix* bark extracts displayed broad-spectrum antiviral activity as it inhibited both enveloped as well as non-enveloped viruses.

**Figure 2:**
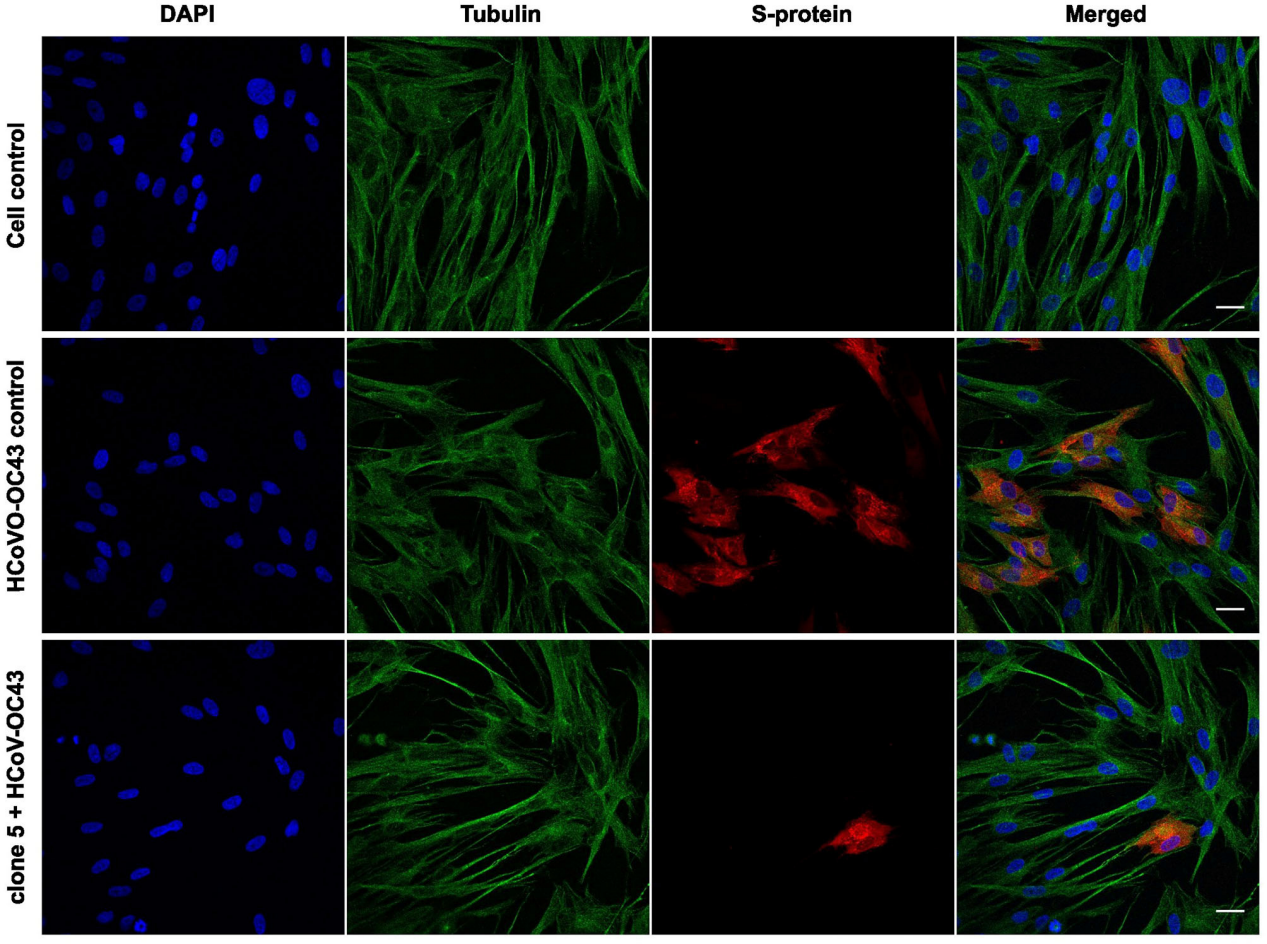
Studying the effect of *Salix* bark extract of clone 5 on HCOV-OC43 using immunofluorescence microscopy. Untreated and treated viruses were added to cells for 1 h at 4°C. After removing the unbound virus, cells were incubated overnight at 34°C before being fixed. The virus was labeled with the spike protein antibody and the cells with the tubulin antibody. Scale bar: 30 μm.

Next, we wanted to observe the effect of willow bark extracts after only one infection cycle. This was studied using confocal microscopy, where the spike (S) protein of the virus was used as a marker of the virion, and cells were labeled using antibodies against tubulin and DAPI stain for the nucleus. The virus was first treated with willow bark extract of clones 5 or 10 for 1 h at 34°C, after which the mixture was added to the cells, and infection was allowed to proceed for 15 h at 34°C. As expected, the mock infection showed no presence of the virus inside the cells, and the spike antibody did not cause any significant background fluorescence. The virus control showed the presence of ample amounts of S-protein inside the cells, confirming successful entry and infection of the virus in 42% of the MRC-5 cells (calculated from 350 cells) (Figure 2). However, when the virus was treated with the bark extract, the infection drastically decreased, and only 1% and 4% (at least 500 cells calculated in total) of MRC-5 cells were infected after treatment with clones 5 or 10, respectively (clone 5 shown in Figure 2). These results support the observation that the willow extract can efficiently decrease the infection of HCoV-OC43 and show in more detail that the virus protein production is halted.

**Figure 3:**
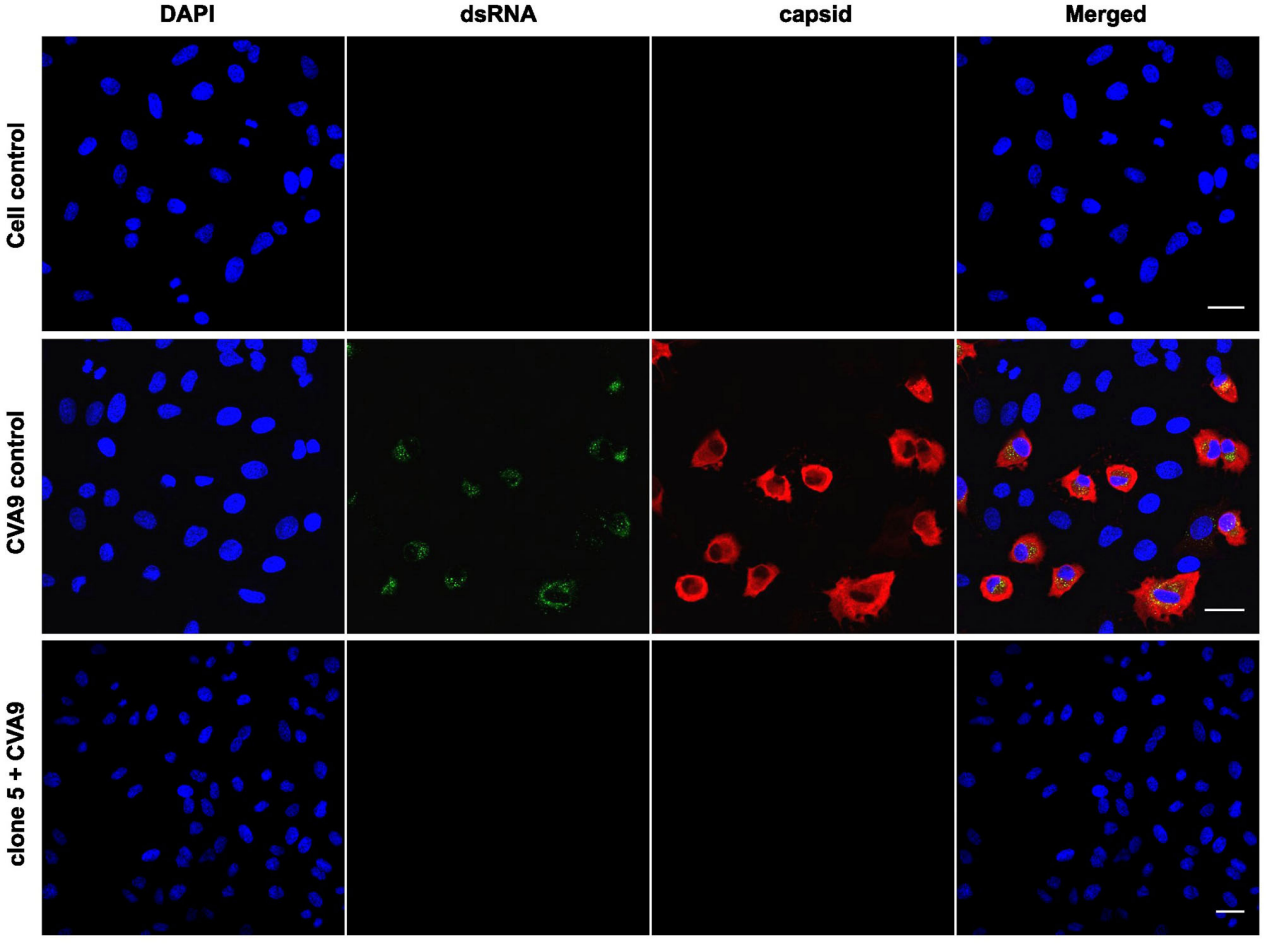
Studying the effect of *Salix* bark extract of clone 5 on CVA9 using immunofluorescence microscopy. Untreated and treated viruses were added to cells for 1 h at 37°C. Afterward, the cells were incubated for 6 h at 37°C before being fixed. The virus was labeled with the capsid protein antibody and the replication intermediate (dsRNA) antibody. Scale bar: 30 μm.

In addition to HCoV-OC43, CVA9 was treated with the willow bark extract, and the infection was followed for one infection cycle (6 h). To evaluate the state of infection, both the viral capsid protein VP1 and the replication intermediate, dsRNA, were immunolabeled, and the results were detected by confocal microscopy. The virus was treated with the extract for 1 h at 37°C, after which the mixture was added to A549 cells, and infection was followed for 6 h. The control virus showed high infection in 37% of the cells (calculated from 500 cells) as the cytoplasm was full of VP1 protein and dsRNA was clearly visible (Figure 3). In contrast, none of the cells were infected in the extract-treated samples (at least 500 cells were calculated) as the signal of both VP1 protein and dsRNA was undetectable (clone 5 is shown in Figure 3).

To better demonstrate the antiviral efficacy against coronavirus, we also performed a virucidal assay. In the assay, HCoV-OC43 was pre-treated with *Salix* bark extract for 15 min at RT, followed by making serial dilutions and adding them to the cells. The result (Table 2) showed an approximately 3–4 log reduction in the virus titer for clones 8 and 10, respectively, compared to the non-treated virus. This demonstrated the exceptional potency of the bark extracts in reducing virus infectivity.

**Table 2:** Quantifying the reduction in virus infectivity using a virucidal assay.

**Sample type**	**Virus titer (PFU/ml)**
Virus control HCoV-OC43	3.88 × 10^10^
HCoV-OC43 treated with clone 8	1.77 × 10^7^
HCoV-OC43 treated with clone 10	1.09 × 10^6^

The virus was treated with bark extract of willow clone 8 or 10 for 1 h, which led to a 3–4 log reduction in virus titer compared to virus control.

Though *Salix* bark extracts worked against the HCoV-OC43, a good surrogate for the more serious and fatal SARS-CoV-2, we wanted to evaluate their antiviral efficacy also against SARS-CoV-2. The antiviral activity of the *Salix* bark extract against SARS-CoV-2 was determined by running a RT-qPCR. Here, the viral RNA was extracted from the supernatant of the infected cells and subjected to quantification by RT-qPCR to determine the presence of viral RNA. Cq values represent the number of PCR cycles taken to exceed the fluorescent intensity threshold line for detecting fluorescent signals from the sample. Cq values are inversely proportional to the amount of presence of viral RNA (cDNA) in the sample. Therefore, the lower the Cq values, the higher the amount of presence of RNA, and vice versa. The virus control had a Cq value of 14.82, suggesting a high amount of viral RNA. However, Cq values increased drastically for viruses treated with *Salix* bark extract of clones 5 and P-16 to 38.33 and 36.90, respectively (Table 3). This indicated that the amount of viral RNA was reduced significantly when it was treated with the willow bark extract. Cq values were also used to calculate the logarithmic reduction in the virus infectivity (Table 3). Based on these calculations, bark extracts caused a 6-log reduction in the viral RNA, which means a very high antiviral effect.

**Table 3:** Cq mean values of the test and virus samples obtained from the qPCR are shown.

**Sample**	**Cq mean value**	**Difference in Cq value compared to VC**	**Log difference**
5	38.3304	23.5093	7.0782
P-16	36.9032	22.0821	6.6475
VC	14.8211	–	–

These mean Cq values were used to calculate the difference between test and virus samples and were further used to calculate the RNA difference. Using the RNA difference, logarithmic reduction was also calculated and is depicted in the table. VC, virus control; P-16 = 2-L scale clone 16.

### 3.2. Effect of time and temperature on the antiviral activity of willow clones

**Figure 4:**
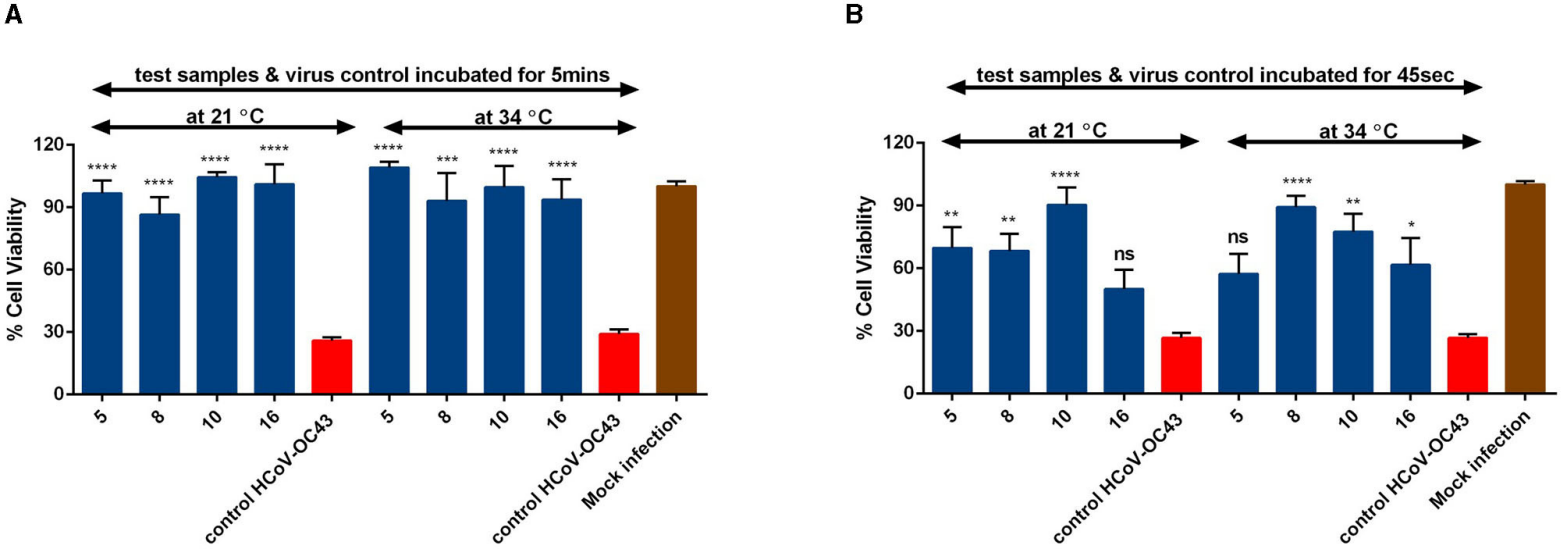
Effect of time and temperature on the antiviral activity of *Salix* bark extracts of selected willow clones (1% v/v) on coronaviruses using CPE inhibition assay. *Salix* bark extract–virus mix was incubated at 34°C and 21°C for **(A)** 5 min and **(B)** 45 s. Virus control and test samples are normalized against the mock infection. The results are the mean of two independent experiments and are shown as average values + standard errors of mean (SEM). *p_ANOVA_ < 0.05, **p_ANOVA_ < 0.01, ***p_ANOVA_ < 0.001, and ****p_ANOVA_ < 0.0001. ns, not significant.

We further wanted to study the impact of different time and temperature on the antiviral efficacy of the *Salix* bark extracts of different willow clones. So, we pre-treated the virus (HCoV-OC43) with the extract for different time periods (5 min and 45 s) and at different temperatures (34°C and RT) before adding it to the cells. When the bark extracts were incubated with the virus at 34°C for 5 min, the extracts were able to protect the cells from virus infection (Figure 4A). The antiviral efficacy was retained even when the 5-min incubation was done at RT (Figure 4A). Remarkably, the extracts were equally effective in blocking the virus infection when the incubation time interval was further reduced to 45 s (Figure 4B). However, a slight reduction in the antiviral activity was evident after this very short incubation time. Overall, the *Salix* bark extracts showed excellent antiviral efficacy against HCoV-OC43 at different conditions.

### 3.3. Time-of-addition studies demonstrate direct action of *Salix* bark extract on coronaviruses

**Figure 5:**
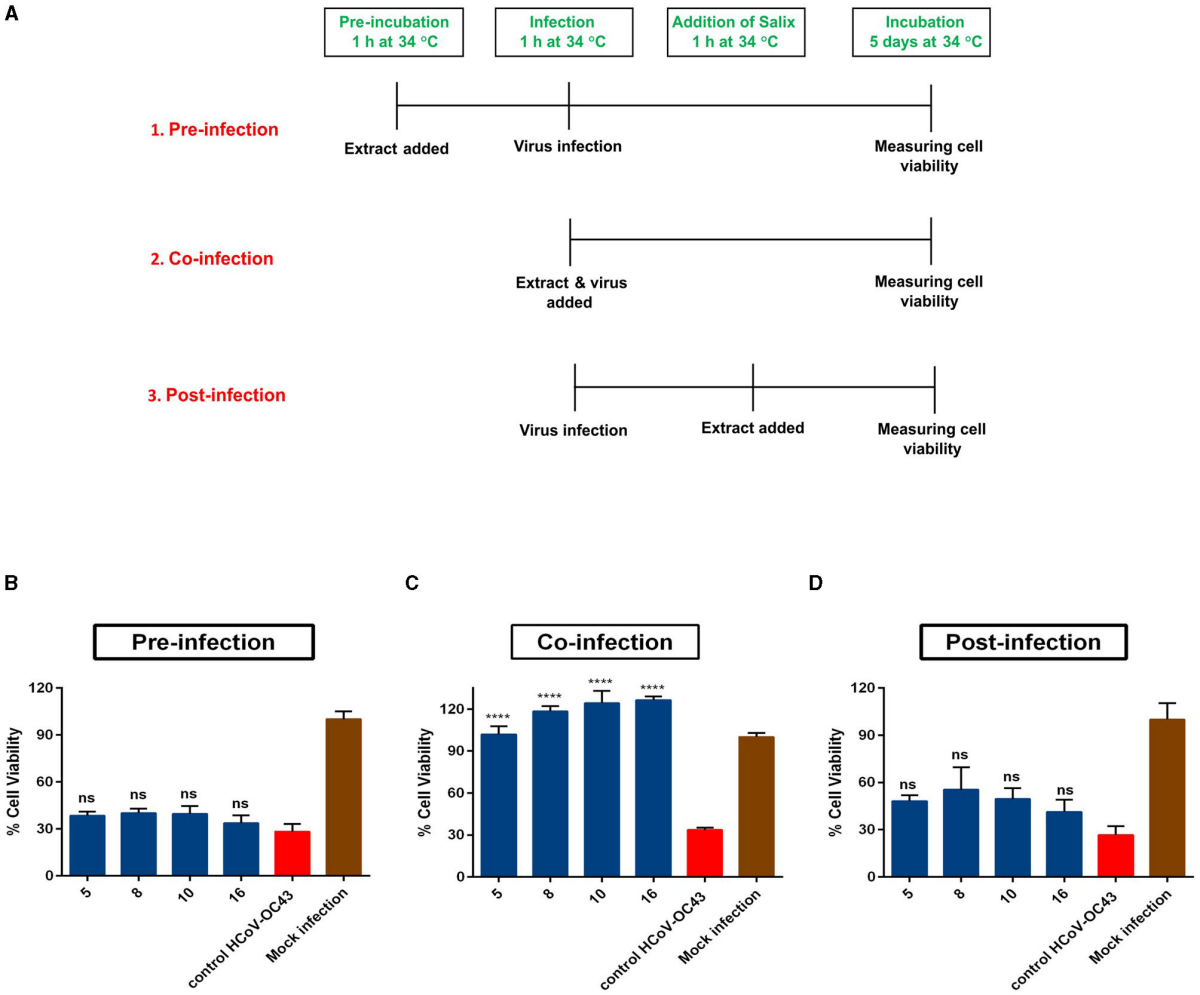
Time-of-addition assay. **(A)** Schematic of three modes of infection studied in the *Salix* bark extract of willow clones (1% v/v) against HCoV-OC43. **(B)** In pre-infection mode, the bark extracts were added 1 h before virus infection. **(C)** Co-infection, where virus and *Salix* clones were added together; **(D)** post-infection mode, where the clones were added 1 h after viral infection. The results are the mean of two independent experiments and are shown as average values + standard error of the mean (SEM). ****p_ANOVA_ < 0.0001. ns, not significant.

To elucidate the mechanism through which *Salix* bark extract blocks the virus infection, we first performed time-of-addition studies. In this assay, three modes of infection were studied (schematic shown in Figure 5A). During pre-infection mode, the extract was added to the cells for 1 h, and after the incubation, the cells were infected with the virus. In case of co-infection, a mix of virus and extract was prepared and added directly to the cells. For the post-infection mode, cells were first infected with the virus for 1 h, after which the extract was added. These studies revealed that when the *Salix* bark extract was added to the cells before or after the virus infection, it was unable to lower the antiviral activity (Figures 5B, D). However, when cells were co-infected with the clone and virus at the same time, the *Salix* bark extract was effective in protecting the cells from HCoV-OC43 infection (Figure 5C). Based on these results, it was evident that the *Salix* bark extracts do not have any effect through the host cells at used concentrations, neither do they interfere with cellular steps of viral infection. Instead, it has a direct effect on the HCoV-OC43 surface and protects the cells from viral infection.

### 3.4. Structural studies with TEM reveal direct effects of *Salix* bark extract treatment on coronaviruses and enteroviruses

**Figure 6:**
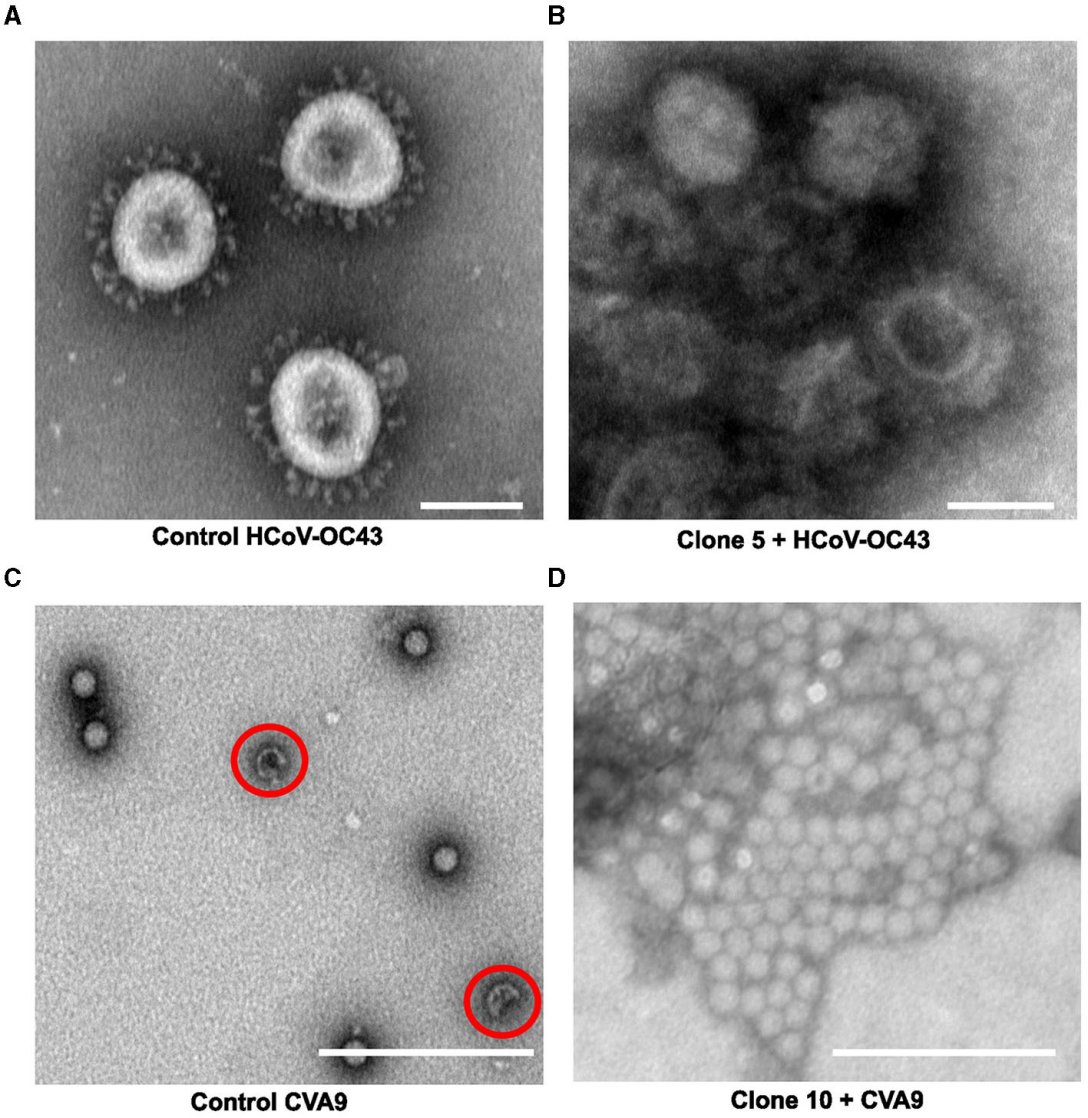
Studying the effect of *Salix* bark extract of clone 5 or 10 on enteroviruses or coronaviruses using transmission electron microscopy. **(A)** control HCoV-OC43, **(B)** HCOV-OC43 treated with clone 5, **(C)** control CVA9, and **(D)** CVA9 treated with clone 10. Using negative staining, empty enterovirus capsids can be identified with a darker center (red circle), whereas intact capsids appear brighter. Negatively stained control coronavirus appeared mostly spherical to elliptical with finger-like projections sticking out from the envelope. A scale bar of 100 nm has been added using Fiji.

The use of negative staining with heavy metal stains along with transmission electron microscopy (TEM) is one of the key imaging techniques that allows the direct visualization of the virus to observe morphological changes the antiviral may cause in the virus structure. To understand the effect of our willow clones on enveloped (OC43) and non-enveloped viruses (CVA9), we pre-treated both viruses with the willow bark extracts and then negatively stained them with 1% phosphotungstic acid. Negatively stained TEM samples of the untreated CVA9, which served as the control virus, showed intact virus particles that have a dark stain around the capsid and a bright center (Figure 6C). Only a small percentage of empty (red circle) capsids were observed. The empty enteroviruses have a darker center due to large openings in the virion and the flow of heavy metal to the inside. The control enteroviruses appeared to be separate from each other and largely spread on the TEM grid. Instead, in the presence of the willow bark extracts, the negatively stained virus samples showed large aggregates of the CVA9 virus, with a dark heavy metal stain around them (Figure 6D). Remarkably, the aggregates did not show dark centers or a darker appearance, strongly suggesting that they stayed intact, like in the virus control.

The negatively stained images of control OC43 showed roughly spherical to elliptical-shaped viruses with patchy dark centers and peplomers (spike proteins) sticking out from the membrane surface (Figure 6A). In addition, the viruses in the control sample were separated from each other on the TEM grid. In contrast, the coronaviruses in the willow bark extract-treated samples appeared to aggregate into clusters (Figure 6B) with a heavy metal stain around them, just like the CVA9. However, what was interesting is that the envelope fringe of the coronavirus looked distorted to some extent, and some of the peplomers had shed from the membrane surface. Also, the inside of the virus appeared to have more stains, suggesting obvious disintegration of the virus structure. These results thus altogether suggest that the willow bark extracts cause aggregation of both non-enveloped and enveloped viruses, increased stability for enteroviruses, and structural disintegration of coronaviruses.

### 3.5. Antiviral effects on enteroviruses are caused by increased stability of the virions (PaSTRy assay)

**Figure 7:**
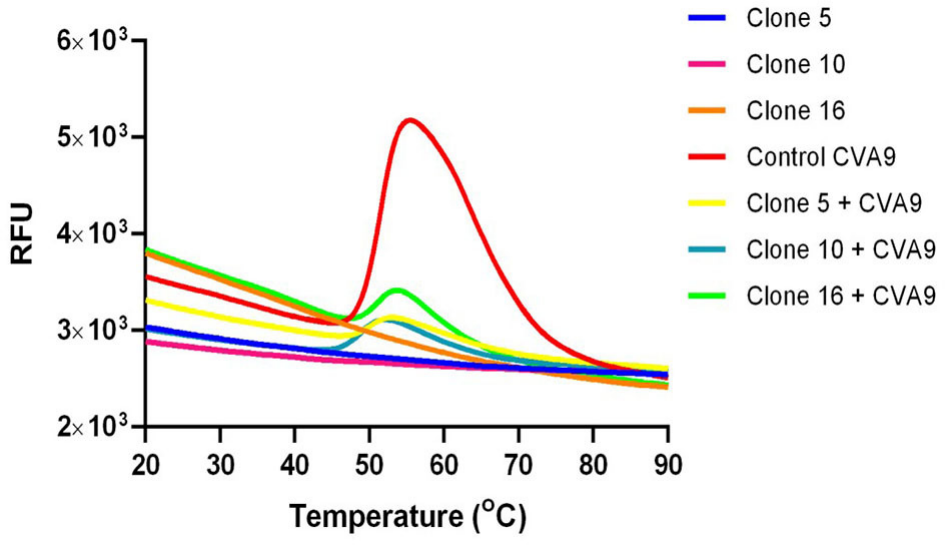
Capsid stability of *Salix* bark extract-treated enteroviruses was studied using the PaSTRy assay. The melt curve is a representation of the increase in fluorescence when the SG II gets access to the viral genome once the virus capsid opens. The red curve is a typical bell-shaped melt curve for enteroviruses. The indigo, pink, and orange curves represent the background fluorescence contributed by the willow clones with SG II. The yellow, blue, and green stunted curves are the melt curves from the virus treated with bark extract from different willow clones. This experiment was performed three times independently, and this is a representative result.

The PaSTRy assay is a good method that is used to study capsid stability among non-enveloped viruses. The assay uses an RNA-binding fluorescent dye (SGII) and a qPCR-based method to determine the melting temperature (Tm) at which the viral genome is released from the capsid. The presence of an inhibitor can typically affect the stability of the capsid by increasing the temperature at which the genome is released. In Figure 7, the red line shows a typical melt curve that is achieved using 1 μg of enterovirus (CVA9). The bell shape of the curve comes from the increase in fluorescence as the capsid proteins unfold during the heating process, making the viral RNA more accessible to the SGII for binding. The melt curve determined by RNA release performed in the presence of the bark extract of different willow clones (yellow, blue, and green colored lines) shows a reduction in the fluorescence peak compared to the control virus. These results suggest that the *Salix* bark extract does not allow the viral capsid to open even at high temperatures and prevents SGII access to the viral genome. When we plotted the fluorescence data as a negative first derivative plot per unit change in temperature, we were able to get the melt peak, which gives us information about the Tm of the virus capsid (data not shown). Interestingly, the Tm results did not differ much between the untreated and treated viruses (for the control virus, the Tm was 51.5°C, and for the virus treated with clones 5, 10, or 16, it was 50°C, 49°C, or 51°C). However, the extent of opening was greatly diminished in the presence of *Salix* bark extracts.

### 3.6. *Salix* extracts do not interfere with the coronavirus binding to the cell surface

**Figure 8:**
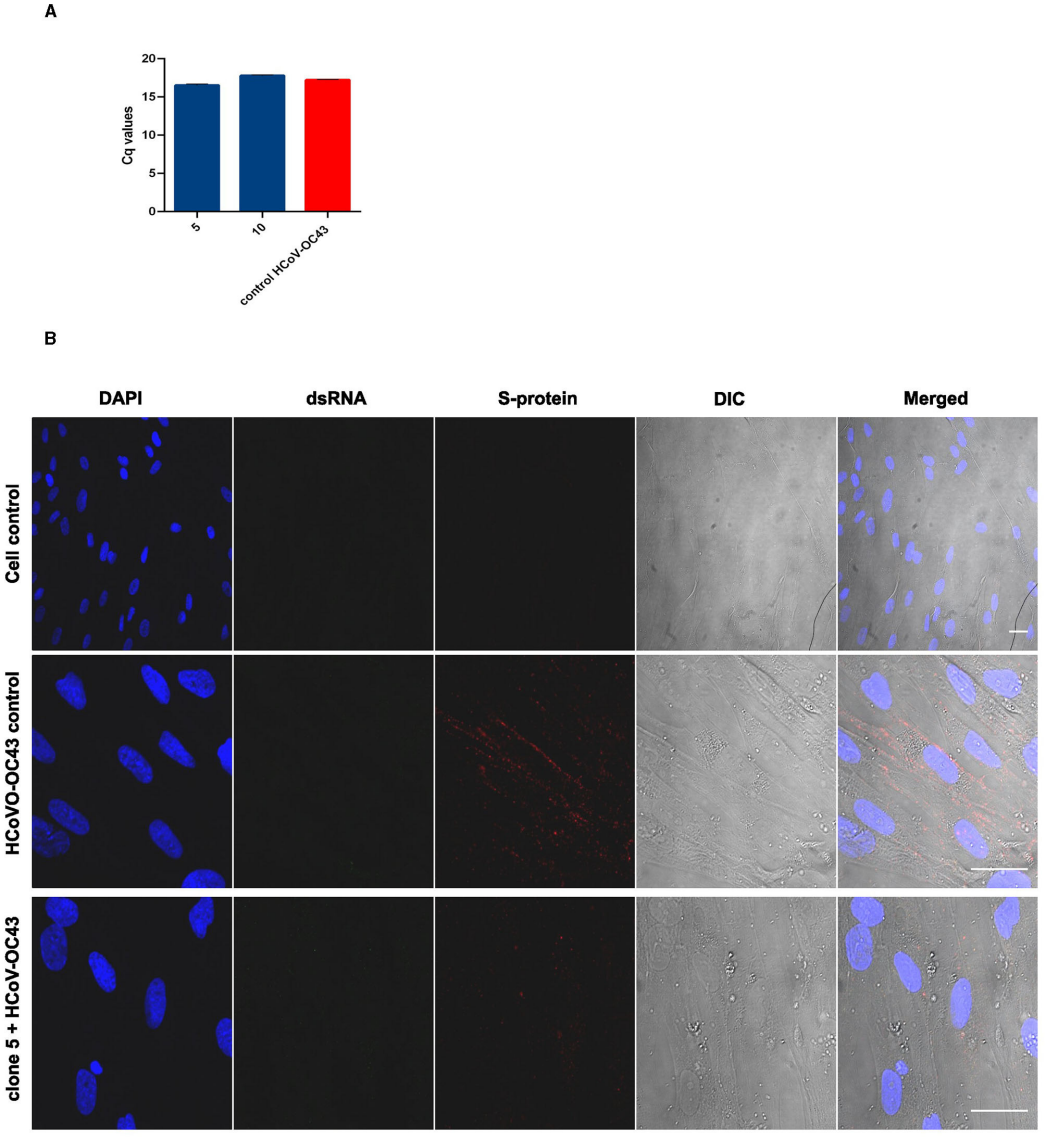
Effect of extract treatment on coronaviruses. **(A)** Studying the effect of *Salix* bark extract of clones 5 and 10 on the binding of HCOV-OC43 to MRC-5 cells. The *Salix*-treated virus was allowed to bind on ice on MRC-5 cells, and the RNA from the virus bound to the cells was isolated, transcribed, and quantified using RT-qPCR. The quantified RNA can be represented by the Cq values from the qPCR. **(B)** Studying the effect of *Salix* bark extract of clone 5 on HCoV-OC43 using immunofluorescence microscopy. Untreated and treated viruses were added to cells for 1 h at RT. After removing the unbound virus, cells were incubated for 1 h at 34°C before being fixed. The virus was labeled with the spike antibody and the replication intermediate (dsRNA) antibody. Scale bar: 30 μm.

The binding assay was designed to study the first step of the coronavirus life cycle, i.e., receptor binding. The experiment was performed by pre-treating the virus with clones 5 or 10 and then adding them to the cells on ice. Ice binding ensures uniform binding and synchronized entry. RNA from the virus bound to its receptor was then isolated and quantified using qPCR. As seen in Figure 8A, the quantification results demonstrated that the extract-treated virus was able to bind to its host cell receptors in a similar fashion as the untreated (control) virus. This indicated that the spikes of coronavirus were intact enough to promote binding to the cell surface.

Confocal studies were also performed to track the movement of the extract-treated coronavirus inside the host cells. For this, spike protein and RNA of the virus were used as markers. The pre-treated virus was added to the cells at RT for 1 h. After removing the unbound virus, cells were incubated for another 1 h at 34°C before washing and fixing them. The aim was to synchronize the virus binding and only provide enough time for its entry. Cells infected with the untreated virus (control HCoV-OC43) showed signal only from the spike protein and not from the RNA (Figure 8B). This confirmed that the virus was able to successfully enter the cells but had not started with its replication step. In addition, the virus was predominantly present in the cellular periphery, where it was accumulated in small vesicles. For cells infected with extract-treated virus, even though the S-protein signal was very faint, the virus appeared in cellular endosomes, similar to that of untreated virus. This gives insight into the fact that the extract-treated virus, after its entry, ends up in endosome vesicles because of which it is not able to continue with its infection.

### 3.7. Chemical composition of hot water extracts

Willow bark extracts were screened by LC-DAD-Orbitrap-MS to characterize their chemical composition. The ion molecular formula of 54 components was defined from the mass spectra, and 37 compounds were tentatively classified (Supplementary Tables S1, S2). Tentative identification was based on measured accurate mass, calculated mass error (<2 ppm), MS^2^ fragmentation, retention time, and wavelength of the UV absorption of the components. Compounds identified were hydroxycinnamic acids (Zhou et al., 2021), salicylates (Pizzato et al., 2022), flavonoids (Panche et al., 2016), flavan-3-ols (Karia et al., 2020), and proanthocyanidin oligomers (Galabov, 2007). Compounds differed qualitatively both between willow species and among genotypes within species.

### 3.8. Stem extract and its fractions

To examine the active components of willow extracts, a pilot-scale willow stem extract was subjected to fractioning in column chromatography over Sephadex LH-20 resin. Fractioning yielded eight fractions. The composition of the stem extract and the fractions was screened by LC-DAD-Orbitrap-MS (Supplementary Figure S1, Table S2), and their efficacy against enterovirus was measured.

Some minor qualitative differences in the content between stem extract and bark extracts of the same species (samples 16 and P-16) were observed (Supplementary Tables S2, S3). Observed differences may be attributable to intraspecific variation among genotypes and to changes in extraction method, extracted plant part (bark vs. whole shoots), harvesting season, growing conditions, and stage of growth.

The stem extract and all the fractions, except fraction 1, were able to protect the A549 cells from CVA9 infection at a concentration of 3 μg/ml (Supplementary Figure S2). A lower concentration of 1 μg/ml had already lost antiviral activity, whereas 5 μg/ml showed very similar results with 3 μg/ml (Supplementary Figure S2). Of the active fractions, fraction 2 contained hydroxycinnamic acid derivatives, such as caffeoyl and coumaroyl quinic acids, and some unidentified compounds. Fractions 3 and 4 consisted of mainly flavonoids, such as quercetin and isorhamnetin glycosides. Fraction 5 contained flavonoids, flavan-3-ols, and procyanidin and prodelphinidin dimers. Fraction 6 had dimeric and trimeric procyanidins and prodelphinidins, together with some unidentified compounds. Fractions 7 and 8 consisted mainly of proanthocyanidins: The total ion chromatogram of fraction 7 showed peaks of trimeric and tetrameric proanthocyanidins together with a hump of higher degree of polymerization proanthocyanidins, while the chromatogram of fraction 8 showed only the unresolved hump of oligomeric proanthocyanidins (Supplementary Figure S1).

All the fractions that contained hydroxycinnamic acids (fraction 2) or polyphenolic flavonoids and proanthocyanidins (fractions 3–8) were active against CVA9 at a concentration of 3 μg/ml. Thus, the fractioning data imply that the polyphenolic structure in general might contribute to the observed activity. However, the active fractions obtained were mixtures of several components, some of them unidentified, and it is possible that the unknown compounds or the synergistic effects between the compounds have an impact on the detected efficacies. The next steps for our studies will be further fractioning of the active preparations and a more detailed investigation of their composition and activities.

## 4. Discussion

Viral outbreaks causing pandemics and yearly epidemics not only affect public health worldwide but also put a strain on the global economy due to the high costs associated with managing these outbreaks. The recent pandemic highlighted the limited resources the world had for fighting such outbreaks. Hence, there is an urgent need for developing broad-spectrum antivirals that can effectively reduce the viral load in the environment and on surfaces. Until 2021, there have been no previous studies exploring the antiviral potential of the *Salix* bark hot water extracts. Our study was the first to report the antiviral properties of these extracts against the highly stable, non-enveloped enteroviruses (CVA9) (Tienaho et al., 2021). In this study, we expanded our research to study the antiviral properties of these extracts against the enveloped human coronaviruses and investigated their mechanism of action against both coronaviruses and enteroviruses.

Our results showed that bark extracts of most of the willow clones were able to protect the MRC-5 cells from HCoV-OC43 infection when the virus was pre-treated with extracts before infecting the cells. Virucidal assay revealed a 3–4 log reduction in the virus infectivity of the extract-treated virus as compared to the untreated virus. The extracts showed efficacy both at high and low temperatures and even after a very short period of incubation (less than a minute). We demonstrated the antiviral nature of the extract against the clinically isolated SARS-CoV-2. All the clones studied were successful in inhibiting the SARS-CoV-2 infection and showed at least a 6-log reduction in the viral RNA.

Interestingly, none of the reference compounds (triandrin, salicin, salicylic acid, and picein) tested showed any antiviral activity against OC43. None of these reference compounds were effective against CVA9 in our previous study (Tienaho et al., 2021). This suggests that any of the commercial reference compounds do not individually contain high enough antiviral activity. This became more evident after fractionation of the bark extract when we observed that all fractions except fraction number 1 contained very high virucidal activities. Those active fractions contained various interesting chemical groups, of which many are known to contain biological activities. The bioactive properties of these bark extracts and broad-spectrum antiviral activity are thus likely to be due to the synergistic effects of the different detected flavonoids, hydroxycinnamic acid derivatives, and procyanidins. This hypothesis is further affirmed by the finding that there seemed to be a strong relationship between the values obtained from the Folin–Ciocalteu test for total phenolics and virucidal results against enteroviruses (Tienaho et al., 2021). Tannins isolated from *Hamamelis virginiana* bark extract have been reported to show antiviral properties against Influenza A virus and Human Papillomavirus (Theisen et al., 2014). Before the outbreak of SARS-CoV-2, researchers had already reported the effectiveness of some polyphenols such as luteolin and resveratrol against SARS-CoV (Yi et al., 2004) and MERS-CoV, respectively (Lin et al., 2017).

Interestingly, significant differences were detected between different willow clones in terms of the antiviral activity of their bark extract. Clones 11 and 12 showed significantly poorer antiviral activity than the rest of the clones. These clones are half-siblings and hybrids between clone 8 as a female parent and the species *S. gmelinii* as a male parent. The poor antiviral activity of these two clones seems to be connected to *S. gmelinii* in the ancestry, but the primary reason remains unclear without more detailed studies on the chemistry of the clones. However, the finding of existing differences between clones is important. The production of willow biomass for any possible antiviral applications would be carried out in plantations of selected willow clones.

Time-of-addition studies demonstrated here that the extracts do not primarily target the viral replication life cycle in the host cells, nor does it have any major intracellular effects. Instead, the studies suggested that the extracts act by interacting directly with the HCoV-OC43 surface. Previously, polyphenols isolated from *Eupatorium perfoliatum* were shown to inhibit influenza virus infection when the virus was treated with the polyphenols before adding it to the cells (Derksen et al., 2016). In another study, tannic acid was reported to inhibit the hepatitis C virus only when added to cells, thus also suggesting that there would be direct action on the virions (Liu et al., 2015). Here, negative staining for TEM showed opposite outcomes for coronaviruses and enteroviruses. While both virions were clustered due to *Salix* extract, the images suggested increased stability for enteroviruses but structural disintegration for coronaviruses. However, the binding assay for coronaviruses pointed out that the extract treatment did not interfere with the binding of the virus. Confocal studies revealed that treated coronaviruses could enter the cells but could not start replication/translation in the cells. Instead, the treated virus ended up inside the cellular endosomes.

Thermal assay clearly demonstrated that the willow bark extract had a stabilizing effect on enteroviruses. Normally, enteroviruses readily release their genome when heated to temperatures between 50°C and 60°C. Strikingly, after treatment with *Salix* bark extract, the genome release was almost totally blocked. An increase in stabilization has been earlier reported for enteroviruses when an antiviral molecule replaces the fatty acid in the hydrophobic pocket of the capsid (Tsang et al., 2000). Enterovirus capsid-binding drugs such as pleconaril often target this hydrophobic pocket, which is normally occupied by an aliphatic fatty acid (Pevear et al., 1999). The hydrophobic pocket is linked with virus stability, and the expulsion of lipid moiety from this pocket is associated with its genome release (Smyth et al., 2003). Also, we showed previously that polyphenols epigallocatechin gallate (EGCG) and resveratrol (RES) effectively inhibit the enterovirus infection by causing clustering and stabilization of the virions and prevent its genome release (Reshamwala et al., 2021). Docking simulations showed that polyphenols can actually bind several sites on the virion, not just the hydrophobic pocket. 4–6 sites were identified depending on the serotype and the compound, which resulted in strong stabilization (Reshamwala et al., 2021). Here, our confocal microscopy studies also confirmed a strong inhibition of infection after 1 h treatment of the virions before adding cells. Even after long periods of incubation, there was no apparent accumulation of enteroviruses in endosomes, suggesting that binding and entry to the cells had been compromised. Analogous to our previous results with EGCG and RES, it is likely that the effective compounds from *Salix* extracts bind strongly directly onto enterovirus capsid, interfere with receptor binding on cells, and inhibit entry to endosomes (Reshamwala et al., 2021).

Future prospects for this study would be a more detailed fractionation and characterization of the *Salix* bark extracts to identify the several bioactive compounds contributing to the antiviral nature of the willow bark. Once their chemical structure has been determined, *in silico* docking studies could be performed to understand the binding sites between the bioactive compounds and the viruses. A computational study to identify different polyphenols isolated from pomegranate peel extract as potential inhibitors for SARS-CoV-2 reported punicalin and punicalagin (two forms of tannins) to interact with the S-protein and to bind with higher affinity to inhibit the viral infection (Suručić et al., 2021). Whether similar or analogous tannins or other antiviral compounds would be found in the *Salix* bark extracts remains to be seen.

## Data availability statement

The raw data supporting the conclusions of this article will be made available by the authors, without undue reservation.

## Author contributions

DR: conceptualization, methodology, data curation, formal analysis, investigation, and writing—original draft. SS: methodology, formal analysis, investigation, and writing—reviewing and editing. JL and MK: data curation, formal analysis, investigation, and writing—reviewing and editing. JT: formal analysis, methodology, and writing—reviewing and editing. ML: methodology and writing—reviewing and editing. PK: writing—reviewing and editing. AV-A: formal analysis and methodology. TJ: conceptualization, project administration, funding acquisition, validation, and writing—reviewing and editing. VM: conceptualization, methodology, investigation, supervision, project administration, funding acquisition, validation, and writing—reviewing and editing. All authors contributed to the article and approved the submitted version.
