# Supplementary material for: Willow (Salix spp.) bark hot water extracts inhibit both enveloped and non-enveloped viruses: study on its anti-coronavirus and anti-enterovirus activities
Source: Front Microbiol. 2023 Nov 8;14:1249794. doi: 10.3389/fmicb.2023.1249794 (PMC10663278; doi:10.3389/fmicb.2023.1249794)
Supplement: Supplementary file 1 [file Data_Sheet_1.docx]

Supplementary Material

**Willow (Salix spp.) bark hot extracts inhibit both enveloped and non-enveloped viruses: study on its anti-coronavirus and anti-enterovirus activities**

Dhanik Reshamwala^1^, Sailee Shroff^1^, Jaana Liimatainen^2^, Jenni Tienaho^2^, Mira Laajala^1^, Petri Kilpeläinen^2^, Anneli Viheriä-Aarnio^2^, Maarit Karonen^3^, Tuula Jyske^2^, Varpu Marjomäki^1*^

^1^Department of Biological and Environmental Sciences/Nanoscience Center, University of Jyväskylä, Jyväskylä, Finland

^2^Natural Resources Institute Finland (Luke), Helsinki, Finland

^3^Natural Chemistry Research Group, Department of Chemistry, University of Turku, Turku, Finland

*** Correspondence:**Varpu Marjomäki
varpu.s.marjomaki@jyu.fi

# Supplementary Data

Supplementary Material should be uploaded separately on submission. Please include any supplementary data, figures and/or tables.

Supplementary material is not typeset so please ensure that all information is clearly presented, the appropriate caption is included in the file and not in the manuscript, and that the style conforms to the rest of the article.

# Supplementary Figures and Tables

Supplementary Table S1. Compounds tentatively identified from hot water extracts of salix clones with LC-DAD-Orbitrap-MS.

Supplementary Table S2. The composition of willow bark extracts determined by LC‒DAD‒Orbitrap-MS.

Supplementary Table S3. The composition of the stem extract and its fractions 1-8 determined by LC‒DAD‒Orbitrap-MS.

## Supplementary Figures


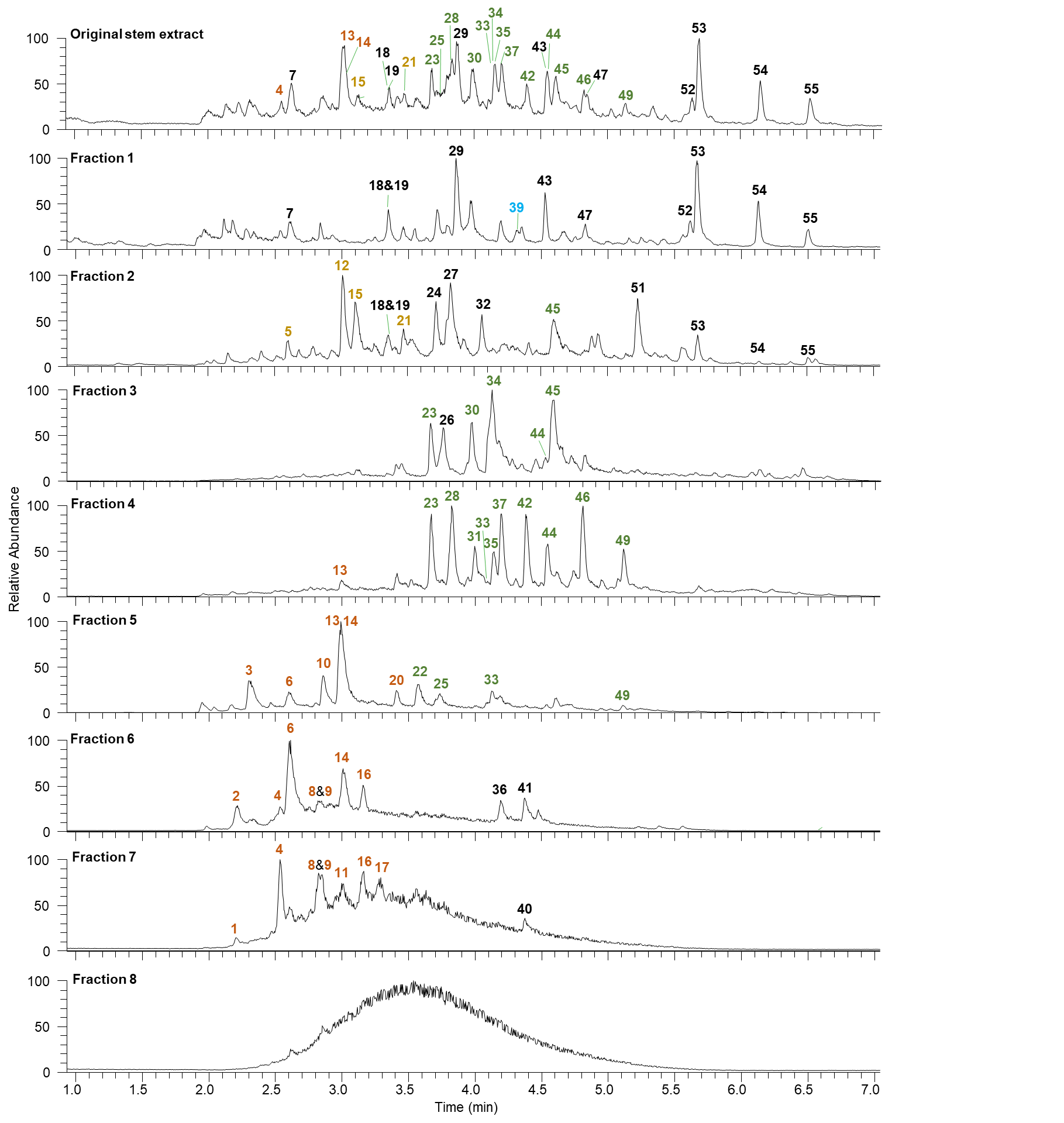


Supplementary Figure S1. Total ion chromatogram in negative ion mode of willow stem extract and its fractions 1-8. Compound numbers refer to Supplementary Table S1. Color of compound number refers to the compound group identified: orange = flavan-3-ols and their oligomers; brownish yellow = hydroxycinnamic acids; green = flavonoids; blue = salicylates; black = unidentified.


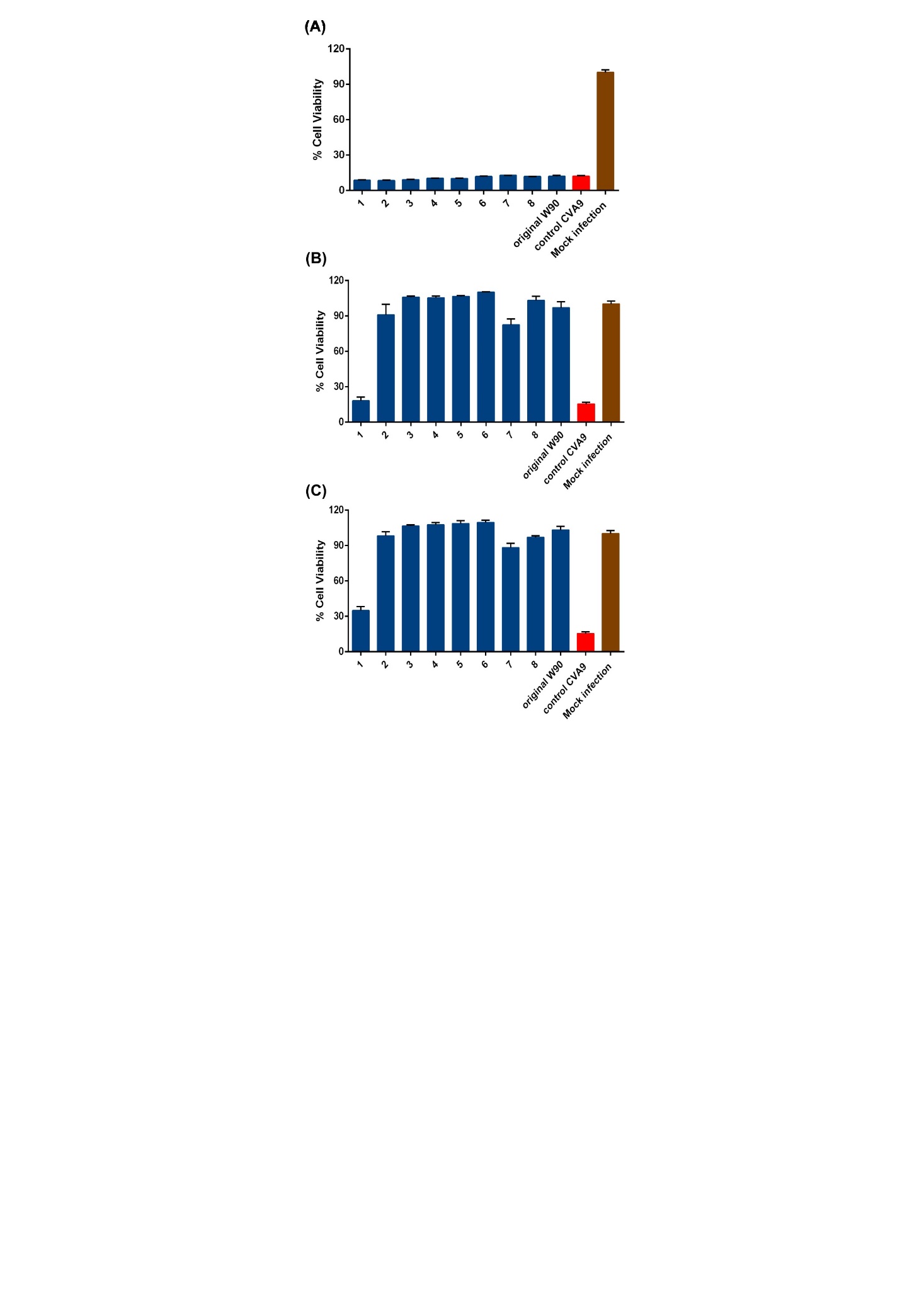


Supplementary Figure S2. Testing antiviral activity of Salix bark extract and its fractions at concentration (A) 1 µg/ml, (B) 3 µg/ml and (C) 5 µg/ml against CVA9 using CPE inhibition assay. Virus control and test samples are normalized against the mock infection. The results are shown as average values + standard error of mean (SEM).
